# An average/deprivation/inequality (ADI) analysis of chronic disease outcomes and risk factors in Argentina

**DOI:** 10.1186/1478-7954-7-8

**Published:** 2009-06-08

**Authors:** Fernando G De Maio, Bruno Linetzky, Mario Virgolini

**Affiliations:** 1 Department of Sociology & Anthropology, Simon Fraser University, Burnaby, British Columbia, Canada; 2 Ministerio de Salud de la Nación, Buenos Aires, Argentina

## Abstract

**Background:**

Recognition of the global economic and epidemiological burden of chronic non-communicable diseases has increased in recent years. However, much of the research on this issue remains focused on individual-level risk factors and neglects the underlying social patterning of risk factors and disease outcomes.

**Methods:**

Secondary analysis of Argentina's 2005 *Encuesta Nacional de Factores de Riesgo *(National Risk Factor Survey, *N *= 41,392) using a novel analytical strategy first proposed by the United Nations Development Programme (UNDP), which we here refer to as the Average/Deprivation/Inequality (ADI) framework. The analysis focuses on two risk factors (unhealthy diet and obesity) and one related disease outcome (diabetes), a notable health concern in Latin America. Logistic regression is used to examine the interplay between socioeconomic and demographic factors. The ADI analysis then uses the results from the logistic regression to identify the most deprived, the best-off, and the difference between the two ideal types.

**Results:**

Overall, 19.9% of the sample reported being in poor/fair health, 35.3% reported not eating any fruits or vegetables in five days of the week preceding the interview, 14.7% had a BMI of 30 or greater, and 8.5% indicated that a health professional had told them that they have diabetes or high blood pressure. However, significant variation is hidden by these summary measures. Educational attainment displayed the strongest explanatory power throughout the models, followed by household income, with both factors highlighting the social patterning of risk factors and disease outcomes. As educational attainment and household income increase, the probability of poor health, unhealthy diet, obesity, and diabetes decrease. The analyses also point toward important provincial effects and reinforce the notion that both compositional factors (i.e., characteristics of individuals) and contextual factors (i.e., characteristics of places) are important in understanding the social patterning of chronic diseases.

**Conclusion:**

The application of the ADI framework enables identification of the regions or groups worst-off for each outcome measure under study. This can be used to highlight the variation embedded within national averages; as such, it encourages a social perspective on population health indicators that is particularly attuned to issues of inequity. The ADI framework is an important tool in the evaluation of policies aiming to prevent or control chronic non-communicable diseases.

## Background

In recent years, the World Health Organization (WHO) has emphasized the substantial worldwide burden of chronic non-communicable diseases (e.g. heart disease, stroke, cancer, respiratory diseases, and diabetes). Their analysis indicates that these diseases account for 60% of the world's deaths, and that close to 80% of these deaths occur in low- and middle-income countries [1]. In part, this represents the success of strategies for controlling infectious diseases and lowering infant mortality, although there is still much to be done in these areas. The WHO projects that the worldwide burden of chronic diseases will substantially increase in the decades to come, and brings to light the implications of this situation; among other consequences, it has adverse macro economic effects by severely diminishing the economic potential of low- and middle-income countries [2,3].

However, although the burden of chronic diseases can be expressed in forgone national income (usually modeled as resulting from loss of productivity due to absenteeism associated with preventable morbidity or premature mortality), or even higher costs to health services [4,5], the importance of this issue is perhaps best understood from a social epidemiological or sociological lens [6]. Epidemiological data demonstrate that chronic diseases account for the majority of unnecessary mortality and morbidity around the world [7]; furthermore, they represent what might be called a 'hidden epidemic' that particularly affects the poor [8,9]. Indeed, analysis of the social patterning of chronic disease outcomes and risk factors emphasizes that the distribution of illness across populations is not random. Morbidity and mortality are socially structured [10]. Chronic non-communicable diseases impose their greatest burdens in conditions of poverty, and contribute to social inequities – inequalities that are avoidable, unnecessary, and unfair [11], creating substantial strain on health and welfare systems.

Understanding of the social burden of chronic diseases has been hampered by epidemiology's traditional concern with individual-level risk factors [12,13]; for example, whether or not someone smokes, or if they exercise, or what kind of diet they consume – an atomistic analysis that in many ways ignores social context. Whilst research has and continues to document a variety of pathways linking these kinds of risk factors to a number of disease outcomes, work on the social determinants of health significantly alters the explanations underlying the patterning of chronic diseases [14] by bringing our attention to the importance of place effects [15]. In many ways, work on the social determinants of health represents a turn to an explicitly sociological understanding of illness, where, following Mills [16], illness – one of the most personal of all personal troubles – is seen in its proper context in relation to wider public issues.

**Figure 1:**
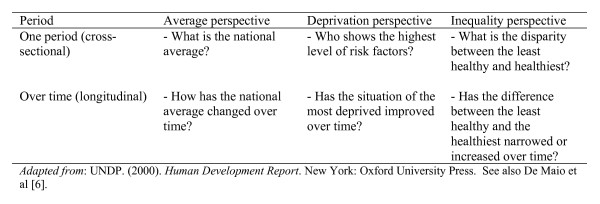
**The ADI framework**.

The social determinants of health perspective overcomes the limitations of a narrow individual-level analysis, but simultaneously emphasizes that recognizing the aggregate burden of chronic diseases is not enough [6,17]. This perspective suggests that data on the social patterning of chronic disease outcomes and risk factors are needed in order to develop effective policy responses. Such data could be used to identify regions, communities, and groups that have a high prevalence of risk factors or suffer from particularly high rates of specific disease outcomes. The 'average/deprivation/inequality' (ADI) framework is useful in this task (see figure 1).

The ADI framework was introduced by the United Nations Development Programme (UNDP) in its 2000 *Human Development Report*. It was originally designed to examine the progressive realization of indicators of human rights and development; for example, the UNDP used the framework to analyze immunization rates in Egypt, literacy rates in India, and under-five mortality rates in Guatemala [18]. To date, it has not been applied to the analysis of chronic non-communicable diseases, yet its simplicity and its ability to model complex underlying social patterns make it a very promising framework for research in this area. Furthermore, consideration of all three components of the framework at one time offers a distinct advantage: it enables analysis of aggregate progress whilst at the same time, brings into focus issues of inequality which are central to current work on the social determinants of health.

Much of the current literature on risk factor data currently falls into the "cross-sectional/average" perspective by reporting national prevalence rates. This is clearly very important, and if repeat cross-sectional or longitudinal surveys are carried out, changes in the national average could be detected. This is a crucial aspect of any attempt to evaluate relevant public policies. However, to understand the social patterning of chronic disease outcomes and risk factors, the second and third steps of the ADI framework are needed. The deprivation perspective seeks to break down the national average by relevant socioeconomic and demographic factors in order to identify the group(s) who experience either the poorest levels of health or the highest levels of risk. In other words, the deprivation perspective seeks to disaggregate national summary statistics by meaningful sociological and/or geographical levels in order to identify the segments of society that experience the heaviest burden. The inequality perspective takes this one step further, by not only identifying the worst-off, but also considering the *difference *between the worst-off and the best-off group. This is particularly important when it comes to public health interventions, which have an unfortunate history of sometimes increasing inequities as an unintended consequence [19].

Building from the ADI framework, this paper examines three inter-related research questions: (1) do chronic disease risk factors and outcomes follow a social gradient in Argentina?; (2) following the deprivation perspective, what parts of Argentine society are most burdened by chronic disease risk factors and outcomes?; and (3) following the inequality perspective, what is the difference between the best-off and worst-off groups? The present analysis focuses primarily on two risk factors (unhealthy diet and obesity) and one related disease outcome (diabetes), a notable health concern in Latin America [4,20].

### What is known about chronic diseases in Argentina?

Argentina is an 'upper-middle-income' country with a Gross Domestic Product per capita of over $14,000 (PPP US$), a combined life expectancy at birth of 74.8 years, and an infant mortality rate of 15 per 1,000 live births [21]. Its population health profile displays a post-epidemiologic transition pattern; data from the WHO [22] indicates that 33.2% of deaths in Argentina are attributable to cardiovascular diseases, 21.2% are attributable to cancers, and 25.5% are attributable to other chronic diseases (in comparison, infectious diseases account for 13.2% and accidents for 6.9%). Such national mortality data indicate the profound importance of chronic diseases as drivers of population health in Argentina [23,24]. Data on age-adjusted disease-specific mortality rates published by the Argentine Ministry of Health provide further evidence of the aggregate burden of chronic diseases in the country [25]. However, until recently, nationally-representative data on the social patterning underlying chronic disease risk factors and outcomes were lacking.

The 2005 *Encuesta Nacional de Factores de Riesgo *(ENFR; National Risk Factor Survey) is the first nationally representative survey carried out on this issue in Argentina. Ferrante and Virgolini [26] have presented the main results from the survey, focusing on the prevalence of the risk factors for cardiovascular diseases, including physical inactivity, consumption of tobacco, raised blood pressure, weight, diet, and heavy alcohol consumption. Their bivariate findings highlight the social patterning of risk factors: "for almost all risk factors assessed, the prevalence was higher in lower income populations, with unmet basic needs and lower educational level" [26]. Consistent with wider patterns of inequity in the country [see [27]], the prevalence of risk factors and self-reported poor health were found to be significantly higher in the northern provinces in comparison to the rest of the country, and congruent with the large literature on social gradients in health, self-reported poor health was found to be significantly related to household income. Building from the results obtained by Ferrante and Virgolini [26], the present study (a) uses multivariate analysis to examine social gradients by socioeconomic characteristics (including household income, educational attainment, employment status, and unsatisfied basic needs) and demographics (sex, age, and marital status), and (b) uses the ADI framework to identify underlying geographical and social patterns.

## Methods

The ENFR is a nationally representative survey with a sample size of 41,392 adults and a response rate of 86.7% [26]. It was carried out in 2005 by Argentina's Ministry of Health in cooperation with the *Instituto Nacional de Estadística y Censos *(INDEC; National Institute of Statistics and Census) and provincial authorities. The survey instrument is based on Step 1 of the WHO's Stepwise Approach [28], a systematic framework for the collection of data on non-communicable diseases. Step 1 is based on a self-reported questionnaire, whilst step 2 includes physical measurements and step 3 also includes biochemical assays. The instrument was validated in Argentina [24] using a variety of approaches. The questionnaire was pilot tested with a sample of 720 households in Tierra del Fuego, with 400 of these households repeating the survey with the same interviewer (to test intra-interviewer reliability) and 200 of these households repeating the survey with a different interviewer (to test inter-interviewer reliability). To ascertain the validity of survey's self-report measures, the pilot test also included physical measurements of height, weight, abdominal circumference, blood pressure and assays of glycemia and cholesterol. Details of the pilot test are presented by Argentina's Ministry of Health [29] and indicate high association between self-reported data and objective measures.

Like similar risk factor surveys that have recently been carried out in a number of countries in Latin American and the Caribbean, the ENFR collected data on a range of data on "common modifiable risk factors" (e.g. unhealthy diet, physical inactivity, tobacco use), "intermediate risk factors" (e.g. raised blood pressure, raised blood glucose, overweight/obesity), chronic diseases, along with self-reported health status, health care service utilization, and socioeconomic/demographic characteristics (for a discussion of common modifiable risk factors, intermediate risk factors, and disease outcomes, see [1]). All data were collected via face-to-face interviews in respondents' homes. The ENFR is expected to play an important role in the development and evaluation of policy aimed at the prevention and control of chronic, non-communicable diseases. Methodological details have been reported by Ferrante and Virgolini [26] and are available from the Ministry of Health's website .

### Demographic and Socioeconomic Measures

Along with standard demographic variables (sex, age, and marital status), our analysis used four measures of socioeconomic status: employment status (employed, unemployed, or not active in the formal labour market), educational attainment, household income, and household unmet basic needs. Educational achievement is recorded in the ENFR dataset using eight categories, ranging from no formal schooling to having graduated from university. These have been recoded into high (attended a post-secondary institution), medium-high (completed or at least attended secondary), medium-low (completed primary schooling), or low (did not complete primary schooling or has not had a formal education) for the purposes of these analyses. Household income (expressed in hundreds of pesos per month) is a continuous variable, and following the practice of Cohen et al [30], was centered around its mean before inclusion in the regression models. Results from preliminary models which used income as a categorical variable revealed linear trends. Unsatisfied basic needs (UBN, or *Necesidades Básicas Insatisfechas*) is a dummy variable that identifies households with critical manifestations of poverty. Based on data collected in the ENFR, it is possible to identify households which (a) lack sufficient dwelling space (defined as more than 3 people per room), (b) contain inadequate housing/building material (e.g. dirt floor), (c) lack proper sanitary conditions (e.g. a working toilet), or (d) contain school-age children (6–12 years) who are not enrolled in school. UBN is a widely-used measure of absolute poverty in Argentina [31-33] and other countries in Latin America [34,35].

These measures of socioeconomic status were chosen on the basis of recent work on the social determinants of health [10] and previous work on health inequalities in Argentina [36]. Together, they can be used to examine what Link and Phelan [14] described as 'fundamental causes' of disease: "... a fundamental cause involves access to resources, resources that help individuals avoid diseases and their negative consequences through a variety of mechanisms. Thus, even if one effectively modifies intervening mechanisms or eradicates some diseases, an association between a fundamental cause and disease will reemerge. As such, fundamental causes can defy efforts to eliminate their effects when attempts to do so focus solely on the mechanisms that happen to link them to disease in a particular situation."

### Health Measures

Four inter-related measures of health are explored in these analyses: self-reported health status, the quality of diet consumed in the past week (a common modifiable risk factor), obesity (an intermediate risk factor), and diabetes. Self-reported health status is widely used in medical sociology and social epidemiology, and has been found to be highly predictive of actual health status, including subsequent morbidity [37] and mortality [38,39]. Although some researchers have raised questions regarding the validity [36,40] and reliability [41] of self-reported health status measures, they remain an important and useful part of the methodological toolbox for inequality researchers and have been used in the context of Latin America [17,42]. An unhealthy diet was conservatively operationalised as not having eaten any fruits or vegetables in at least five days during the week preceding the interview. Following standard practice, obesity was operationalised as a body mass index (BMI) of 30 kg/m^2 ^or greater. Our diabetes measure relies on a question in the survey which asked respondents if at any time a doctor, nurse or other medical professional had told them they had diabetes or high blood sugar. Similar operationalisation strategies were used by Fleischer et al [43], in their analysis of ENFR data for Buenos Aires.

### Statistical Analysis

All analyses were conducted using Stata's survey analysis commands and weighted using survey sampling weights [44]. Associations between categorical variables were ascertained with chi-square tests of significance. Logistic regression analyses were carried out in three steps: first, a series of unadjusted models examine the bivariate relationship between each independent variable and the dependent variable. Step two considers the effects of the combined set of socioeconomic characteristics, and step three adds demographic variables. The reference person for the fully adjusted model is a married man aged 44 (the mean age of this sample) who has completed at least some post-secondary education, is employed, enjoys an average level of household income, and lives in the Federal Capital of Buenos Aires. All of the dependent variables were coded as 0 or 1 (following standard practice in the literature, self-reported health status was recoded 0 = excellent, very good, or good and 1 = fair or poor). The results are interpreted with odds ratios and 95% confidence intervals [19,45]. Plots of predicted probabilities were generated using the method of Long and Freese [46]; their technique enables the computation of predicted values when one independent variable varies and others are held constant. In these analyses, household income (a continuous variable) was chosen as the varying independent variable. Other characteristics (i.e. other values for independent variables) where chosen on the basis of the ADI framework to identify the worst-off and best-off groups in each of the models.

## Results

The demographic and socioeconomic characteristics of the sample are described in table 1. A slight majority of respondents are women and the modal age group is 35 – 49. The majority of respondents are married and employed. The modal educational attainment is medium-high (completed or at least attended secondary), whilst 12.8% of respondents either did not complete primary schooling or had no formal education. At least one unsatisfied basic need was experienced by 17.0% of respondents. Mean household income was $860 (pesos per month, with a maximum of $5,500 and a standard deviation of $816).

**Table 1:** Demographic profile of the sample

	N	% (Weighted)
Demographic variables		
*Sex*		
Male	17,827	47.5%
Female	23,565	52.5%
		
*Age*		
18 – 24	5,957	18.1%
25 – 34	9,059	20.2%
35 – 49	11,714	25.9%
50 – 64	8,267	21.0%
65+	6,395	14.8%
		
*Marital status*		
Married	22,501	60.5%
Separated or divorced	4,143	7.2%
Widowed	4,019	7.8%
Single	10,729	24.5%
		
Socioeconomic characteristics		
*Employment status*		
Employed	26,174	62.8%
Unemployed	2,070	5.5%
Not active	13,148	31.7%
		
*Education*		
High	10,842	24.0%
Medium-high	15,002	37.0%
Medium-low	9,672	26.3%
Low	5,819	12.8%
		
*Unsatisfied basic needs*		
At least one unsatisfied basic need	6,337	17.0%
No unsatisfied basic needs	35,505	83.0%
		
	Mean	Standard Deviation
Household income (pesos per month)	860	816

*Notes*: Age was recorded in the ENFR dataset as a continuous variable. For the purposes of this descriptive table, it has been categorized into five groups. The multivariate analysis uses age as a continuous variable centered on its mean of 44 years.

Overall, 19.9% of the sample reported being in poor/fair health, 35.3% reported not eating any fruits or vegetables in five days of the week preceding the interview, 14.7% had a BMI of 30 or greater, and 8.5% indicated that a health professional had told them that they have diabetes or high blood pressure (see table 2). The social patterning underlying our measures of health status is also explored in table 2, where the association between each dependent variable and educational attainment is tested. A statistically significant gradient-like relationship is observed between educational attainment and self-rated health status, with respondents with lower educational attainment reporting higher rates of 'poor' or 'fair' health status (χ^2 ^= 5198, p < 0.001). Similar gradients are observed with the quality of diet in the week preceding the interview (χ^2 ^= 342, p < 0.001), weight, and diabetes. The social patterning of obesity rates is particularly striking in these data, with the percentage of respondents with a BMI of 30 or more being 8.9% in the high education group and 21.4% in the low education group (χ^2 ^= 1136, p < 0.001). Similarly, the percentage of respondents with diagnosed diabetes ranges from 5.1% in the high education group to 14.7% in the low education group (χ^2 ^= 594, p < 0.001).

**Table 2:** Health status by educational attainment (*N *and weighted percentages)

	N	Educational Attainment				Overall
		High	Medium-high	Medium-low	Low	
Self-reported health status *						
Excellent	3,647	16.6	9.7	6.5	3.3	9.2
Very good	10,158	42.3	26.2	15.5	11.3	25.4
Good	18,141	34.7	48.6	50.9	45.9	45.5
Fair	8,260	7.8	13.8	23.5	32.1	17.3
Poor	1,129	0.6	1.6	3.6	7.4	2.6
						
Diet in the past week *						
Consumed fruits and vegetables	12,743	35.8	28.8	27.9	26.0	29.9
Consumed some fruits but no vegetables	5,184	13.2	13.1	13.0	14.2	13.2
Consumed some vegetables but no fruit	10,036	22.2	20.9	21.1	24.2	21.7
Consumed neither fruit nor vegetables in five days of the last week	13,372	28.9	37.2	38.1	35.6	35.3
						
Obesity *						
BMI < 25	18,740	61.7	53.1	42.3	38.8	50.9
25 = < BMI <30 (overweight)	13,161	29.4	33.2	38.9	39.9	34.5
BMI > = 30 (obese)	6,009	8.9	13.7	18.8	21.4	14.7
						
Diabetes *						
Yes	3,670	5.1	6.5	11.4	14.7	8.5
No	37,494	94.5	93.5	88.6	85.4	91.5

*Notes*: * Chi-square test significant at p < 0.001.

The cross-tabulations above indicate the presence of a significant social gradient, as measured by differences in educational attainment. These relationships are explored in greater detail below using logistic regression.

There is a clear social patterning underlying self-reported health status (see steps A1 – A3 in table 3). The significant bivariate association observed in table 2 is likewise detected in the logistic regression models, with the odds of reporting poor health significantly increasing as educational attainment decreases. This gradient pattern remains significant after the inclusion of other socioeconomic and demographic characteristics (step A3). Household income also reinforces this pattern, both in terms of the unadjusted model (step A1) and the adjusted models (steps A2 and A3), with each 100 pesos per month in household income decreasing the likelihood of reporting poor health by 5%. UBN is a strong predictor of poor self-rated health in the unadjusted model (OR = 1.37, 95% CI = 1.19 – 1.58), but its significance diminishes with the inclusion of other socioeconomic factors. Women and respondents who rely on the public sector for healthcare have a significantly higher risk of poor health. Ten of the twenty-three provincial dummy variables are significant in step A3; two of these provinces (Buenos Aires and La Pampa) have ORs (odds ratios) less than 1.00 and eight provinces have ORs significantly greater than 1.00. Respondents from the northwestern province of Jujuy (OR = 2.35, 95% CI = 1.77 – 3.14) stand out as being associated with a substantially greater likelihood of poor self-reported health.

**Table 3:** Logistic regression analyses for predictors of poor self-rated health and unhealthy diet

	Self-rated health						Unhealthy diet					
	Step A1		Step A2		Step A3		Step B1		Step B2		Step B3	
	OR	95% CI	OR	95% CI	OR	95% CI	OR	95% CI	OR	95% CI	OR	95% CI
Socioeconomic characteristics												
Household income	0.92	0.91 – 0.94	0.95	0.94 – 0.97	0.95	0.94 – 0.97	0.98	0.97 – 0.98	0.99	0.98 – 0.99	0.98	0.98 – 0.99
												
*UBN*												
No	1.00	-	1.00	-	1.00	-	1.00	-	1.00	-	1.00	-
Yes	1.37	1.19 – 1.58	0.85	0.72 – 1.00	1.14	0.95 – 1.36	1.51	1.32 – 1.74	1.19	1.02 – 1.39	1.04	0.88 – 1.23
												
*Educational* *attainment*												
High	1.00	-	1.00	-	1.00	-	1.00	-	1.00	-	1.00	-
Medium-high	2.00	1.67 – 2.39	1.76	1.46 – 2.12	1.51	1.24 – 1.84	1.46	1.29 – 1.64	1.18	1.04 – 1.35	1.24	1.08 – 1.43
Medium-low	4.09	3.42 – 4.89	3.08	2.54 – 3.74	1.91	1.54 – 2.38	1.51	1.32 – 1.72	1.22	1.05 – 1.43	1.61	1.36 – 1.91
Low	7.19	5.93 – 8.72	4.66	3.75 – 5.80	2.34	1.82 – 3.02	1.36	1.16 – 1.59	0.99	0.82 – 1.19	1.57	1.27 – 1.94
												
*Employment* *status*												
Employed	1.00	-	1.00	-	1.00	-	1.00	-	1.00	-	1.00	-
Unemployed	1.42	1.09 – 1.85	1.25	0.94 – 1.65	1.33	1.00 – 1.78	1.06	0.85 – 1.32	0.95	0.76 – 1.20	0.96	0.76 – 1.22
Not active	2.40	2.15 – 2.68	1.85	1.64 – 2.08	1.31	1.14 – 1.50	0.70	0.63 – 0.77	0.70	0.63 – 0.79	0.95	0.83 – 1.07
												
**Reliant on** **public sector** **for healthcare**												
No	1.00	-	1.00	-	1.00	-	1.00	-	1.00	-	1.00	-
Yes	1.36	1.21 – 1.52	0.95	0.83 – 1.09	1.38	1.19 – 1.60	1.67	1.51 – 1.85	1.42	1.26 – 1.59	1.24	1.09 – 1.41
												
**Demographic** **characteristics**												
*Sex*												
Male	1.00	-			1.00	-	1.00	-			1.00	-
Female	1.43	1.29 – 1.60			1.34	1.18 – 1.53	0.62	0.57 – 0.69			0.62	0.55 – 0.69
												
*Age*	1.04	1.04 – 1.04			1.04	1.03 – 1.04	0.98	0.98 – 0.98			0.98	0.97 – 0.98
												
*Marital* *status*												
Married	1.00	-			1.00	-	1.00	-			1.00	-
Separated or divorced	1.11	0.92 – 1.33			0.90	0.74 – 1.11	1.06	0.89 – 1.26			1.15	0.95 – 1.39
Widowed	2.15	1.83 – 2.53			0.65	0.53 – 0.79	0.61	0.50 – 0.74			1.03	0.82 – 1.30
Single	0.53	0.45 – 0.62			0.85	0.71 – 1.00	1.41	1.26 – 1.58			0.99	0.87 – 1.14
												
*Provinces*												
Buenos Aires (Capital)	1.00	-			1.00	-	1.00	-			1.00	-
Buenos Aires (Province)	1.25	1.02 – 1.54			0.75	0.59 – 0.95	1.37	117 – 1.61			0.91	0.76 – 1.10
Catamarca	1.72	1.37 – 2.16			1.31	1.01 – 1.72	1.76	1.46 – 2.11			1.17	0.95 – 1.44
Córdoba	1.55	1.23 – 1.95			1.11	0.85 – 1.45	1.43	1.19 – 1.73			1.08	0.88 – 1.33
Corrientes	1.48	1.16 – 1.81			0.87	0.67 – 1.14	1.69	1.41 – 2.01			1.04	0.84 – 1.27
Chaco	1.43	1.15 – 1.79			0.85	0.66 – 1.11	1.52	1.28 – 1.80			0.86	0.70 – 1.05
Chubut	1.06	0.83 – 1.34			0.81	0.62 – 1.06	1.10	0.91 – 1.32			0.77	0.62 – 0.95
Entre Ríos	1.35	1.07 – 1.71			0.79	0.59 – 1.05	1.17	0.97 – 1.42			0.78	0.63 – 0.97
Formosa	2.30	1.82 – 2.89			1.29	0.98 – 1.70	0.64	0.52 – 0.79			0.38	0.30 – 0.48
Jujuy	3.08	2.44 – 3.88			2.35	1.77 – 3.14	0.51	0.41 – 0.64			0.31	0.24 – 0.40
La Pampa	0.92	0.71 – 1.17			0.54	0.40 – 0.73	1.32	1.09 – 1.60			0.87	0.70 – 1.08
La Rioja	1.86	1.51 – 2.31			1.56	1.22 – 2.00	1.63	1.37 – 1.93			1.08	0.89 – 1.31
Mendoza	1.19	0.95 – 1.49			0.80	0.62 – 1.03	0.89	0.75 – 1.08			0.67	0.54 – 0.82
Misiones	1.71	1.36 – 2.14			1.02	0.78 – 1.33	0.57	0.46 – 0.70			0.33	0.26 – 0.42
Neuquén	1.73	1.39 – 2.16			1.35	1.04 – 1.74	1.04	0.87 – 1.24			0.69	0.56 – 0.85
Río Negro	1.59	1.27 – 1.99			1.18	0.90 – 1.53	1.26	1.05 – 1.52			0.86	0.70 – 1.06
Salta	2.28	1.83 – 2.85			1.59	1.22 – 2.07	0.69	0.57 – 0.83			0.38	0.30 – 0.48
San Juan	1.80	1.45 – 2.23			1.21	0.94 – 1.57	0.99	0.83 – 1.19			0.64	0.52 – 0.79
San Luis	1.71	1.38 – 2.12			1.21	0.94 – 1.57	1.23	1.03 – 1.47			0.82	0.67 – 1.00
Santa Cruz	1.46	1.17 – 1.84			1.51	1.16 – 1.98	1.71	1.42 – 2.04			1.25	1.01 – 1.53
Santa Fe	1.24	0.99 – 1.56			0.82	0.63 – 1.08	1.27	1.06 – 1.52			0.95	0.77 – 1.16
Santiago del Estero	2.01	1.63 – 2.47			1.32	1.03 – 1.69	1.20	1.01 – 1.42			0.74	0.61 – 0.90
Tucumán	2.11	1.71 – 2.61			1.52	1.18 – 1.96	1.13	0.95 – 1.35			0.72	0.58 – 0.88
Tierra del Fuego	1.09	0.85 – 1.39			1.50	1.13 – 1.99	1.62	1.34 – 1.94			1.25	1.01 – 1.53
												
*N*	38,223 – 41,392		37,718		37,718		38,223 – 41,392		37,718		37,718	

Note: Results in steps A1 and B1 (table 3) and C1 and D1 (table 4) refer to bivariate (unadjusted) odds ratios and confidence intervals.

Similar relationships can be observed with relation to having an unhealthy diet, with educational attainment and household income remaining significant predictors (see table 3). UBN is a strong predictor in the unadjusted model (OR = 1.51, 95% CI = 1.32 – 1.74) but loses its significance in the fully adjusted model. Respondents who rely on the public sector for healthcare are more likely to not having eaten fruits or vegetables in five days of the week preceding the interview (see steps B1 – B3). Employment status and marital status do not display much explanatory power, whilst sex and age are significant predictors. Fourteen provincial dummies display significant effects, with respondents from the southern provinces of Tierra del Fuego (OR = 1.25, 95% CI = 1.01 – 1.53) and Santa Cruz (OR = 1.02, 95% CI = 1.01 – 1.53) having an increased probability of not having eaten fruits or vegetables.

Educational attainment remains a consistently significant predictor of obesity and diabetes (see table 4). Indeed, the relationships continue to display gradient-like patterns, with respondents in the low educational group experiencing the greatest risk of obesity (OR = 1.54, 95% CI = 1.17 – 2.02), followed by the medium-low (OR = 1.47, 95% CI = 1.18 – 1.83), and medium-high (OR = 1.37, 95% CI = 1.14 – 1.65) groups, in contrast to the high educational group. Household income supports this gradient pattern, with a 1% reduction in likelihood of obesity with each additional 100 pesos of monthly income. UBN, employment status, and sex do not display explanatory power in these models. Ten provincial dummies have significant effects; all of them are positive, indicating an increased likelihood of obesity.

**Table 4:** Logistic regression analyses for predictors of obesity and diabetes

	Obesity						Diabetes					
	Step C1		Step C2		Step C3		Step D1		Step D2		Step D3	
	OR	95% CI	OR	95% CI	OR	95% CI	OR	95% CI	OR	95% CI	OR	95% CI
Socioeconomic characteristics												
Household income	0.99	0.98 – 0.99	1.00	0.99 – 1.01	0.99	0.98 – 1.00	0.98	0.97 – 0.99	1.00	0.99 – 1.01	0.99	0.98 – 1.01
												
*UBN*												
No	1.00	-	1.00	-	1.00	-	1.00	-	1.00	-	1.00	-
Yes	1.05	0.87 – 1.27	0.84	0.68 – 1.03	0.96	0.78 – 1.20	0.83	0.64 – 1.07	0.80	0.60 – 1.05	1.12	0.83 – 1.50
												
*Educational* *attainment*												
High	1.00	-	1.00	-	1.00	-	1.00	-	1.00	-	1.00	-
Medium-high	1.63	1.39 – 1.92	1.64	1.37 – 1.96	1.37	1.14 – 1.65	1.31	1.05 – 1.63	1.53	1.22–1.92	1.21	0.96 – 1.54
Medium-low	2.38	2.00 – 2.82	2.28	1.87 – 2.78	1.47	1.18 – 1.83	2.39	1.89 – 3.03	2.51	1.94 – 3.25	1.34	1.02 – 1.77
Low	2.79	2.28 – 3.41	2.60	2.06 – 3.29	1.54	1.17 – 2.02	3.20	2.47 – 4.15	3.16	2.34 – 4.26	1.42	1.01 – 1.99
												
*Employment* *status*												
Employed	1.00	-	1.00	-	1.00	-	1.00	-	1.00	-	1.00	-
Unemployed	0.73	0.53 – 1.00	0.66	0.47 – 0.93	0.75	0.53 – 1.05	0.65	0.44 – 0.97	0.56	0.38 – 0.82	0.60	0.41 – 0.90
Not active	1.17	1.02 – 1.34	1.07	0.93 – 1.25	0.97	0.82 – 1.16	2.20	1.87 – 2.59	1.81	1.52 – 2.16	1.36	1.11 – 1.68
												
**Reliant on** **public sector** **for healthcare**												
No	1.00	-	1.00	-	1.00	-	1.00	-	1.00	-	1.00	-
Yes	1.01	0.88 – 1.17	0.85	0.72 – 1.00	1.03	0.87 – 1.23	0.81	0.67 – 0.97	0.77	0.63 – 0.95	1.12	0.89 – 1.40
												
**Demographic** **characteristics**												
*Sex*												
Male	1.00	-			1.00	-	1.00	-			1.00	-
Female	0.89	0.79 – 1.01			0.97	0.84 – 1.12	1.11	0.95 – 1.31			1.00	0.83 – 1.19
												
*Age*	1.02	1.02 – 1.03			1.02	1.01 – 1.02	1.04	1.04 – 1.04			1.04	1.03 – 1.04
												
*Marital* *status*												
Married	1.00	-			1.00	-	1.00	-			1.00	-
Separated or divorced	0.75	0.58 – 0.96			0.69	0.53 – 0.89	0.89	0.67 – 1.17			0.92	0.67 – 1.26
Widowed	1.07	0.87 – 1.32			0.71	0.55 – 0.91	1.76	1.40 – 2.20			0.67	0.51 – 0.88
Single	0.30	0.25 – 0.36			0.44	0.36 – 0.54	0.37	0.29 – 0.46			0.62	0.49 – 0.79
												
*Provinces*												
Buenos Aires (Capital)	1.00	-			1.00	-	1.00	-			1.00	-
Buenos Aires (Province)	1.28	1.01 – 1.62			1.10	0.85 – 1.43	1.09	0.83 – 1.44			0.91	0.67 – 1.23
Catamarca	1.79	1.38 – 2.32			1.74	1.30 – 2.32	1.11	0.82 – 1.51			1.16	0.82 – 1.63
Córdoba	1.28	0.96 – 1.70			1.21	0.88 – 1.65	1.31	0.95 – 1.79			1.26	0.89 – 1.78
Corrientes	1.43	1.10 – 1.85			1.29	0.96 – 1.74	0.98	0.72 – 1.34			0.95	0.67 – 1.36
Chaco	1.33	1.03 – 1.71			1.13	0.84 – 1.53	1.10	0.82 – 1.47			1.06	0.75 – 1.50
Chubut	1.50	1.16 – 1.94			1.26	0.95 – 1.67	1.19	0.89 – 1.59			1.22	0.89 – 1.68
Entre Ríos	1.21	0.92 – 1.59			0.99	0.73 – 1.34	1.00	0.73 – 1.38			0.82	0.57 – 1.17
Formosa	1.67	1.25 – 2.22			1.51	1.08 – 2.11	1.03	0.74 – 1.44			0.94	0.63 – 1.39
Jujuy	1.32	0.99 – 1.75			1.28	0.93 – 1.75	0.49	0.35 – 0.68			0.47	0.32 – 0.68
La Pampa	1.42	1.07 – 1.88			1.12	0.82 – 1.53	0.92	0.66 – 1.28			0.88	0.61 – 1.26
La Rioja	1.56	1.22 – 2.00			1.59	1.21 – 2.09	1.19	0.89 – 1.59			1.25	0.90 – 1.73
Mendoza	1.48	1.15 – 1.91			1.31	0.99 – 1.73	0.81	0.60 – 1.11			0.73	0.52 – 1.02
Misiones	1.08	0.82 – 1.42			0.93	0.68 – 1.27	0.99	0.73 – 1.36			0.89	0.62 – 1.28
Neuquén	1.45	1.13 – 1.86			1.31	0.99 – 1.74	1.16	0.86 – 1.56			1.26	0.90 – 1.76
Río Negro	1.61	1.23 – 2.09			1.35	1.00 – 1.81	1.27	0.94 – 1.71			1.13	0.81 – 1.57
Salta	1.23	0.93 – 1.62			1.20	0.88 – 1.63	0.51	0.37 – 0.71			0.48	0.33 – 0.70
San Juan	1.68	1.31 – 2.16			1.48	1.11 – 1.97	1.17	0.87 – 1.57			1.11	0.79 – 1.54
San Luis	1.39	1.08 – 1.79			1.23	0.93 – 1.63	1.24	0.93 – 1.66			1.23	0.88 – 1.70
Santa Cruz	2.07	1.61 – 2.67			2.06	1.56 – 2.73	1.04	0.76 – 1.42			1.10	0.77 – 1.57
Santa Fe	1.55	1.19 – 2.01			1.35	1.01 – 1.80	1.09	0.80 – 1.48			0.91	0.64 – 1.28
Santiago del Estero	1.49	1.16 – 1.91			1.34	1.01 – 1.76	1.17	0.88 – 1.56			1.13	0.81 – 1.56
Tucumán	1.60	1.24 – 2.06			1.55	1.16 – 2.06	0.86	0.63 – 1.16			0.84	0.59 – 1.19
Tierra del Fuego	2.14	1.67 – 2.76			2.14	1.62 – 2.83	1.20	0.88 – 1.64			1.54	1.09 – 2.18
												
*N*	35,124 – 37,955		34,665		34,665		38,067 – 41,219		37,566		37,566	

The social patterning underlying the diagnosis of diabetes shows the continued importance of educational attainment as a predictive factor. Throughout steps D1 to D3 (table 4), educational attainment shows a gradient-like pattern, with the probability of a diagnosis for diabetes increasing at lower levels of education. Household income effects on the whole are not significant (with the exception of the unadjusted model, which is consistent with the gradient pattern shown by education). Employment status displays a mixed effect, with unemployment associated with a decreased probability of diabetes in both the unadjusted and adjusted models, and not being active in the labour market associated with an increased probability. Only three provincial dummies are statistically significant, with respondents from the Northwestern provinces of Jujuy (OR = 0.47, 95% CI = 0.32 – 0.68) and Salta (OR = 0.48, 95% CI = 0.33 – 0.70) displaying a decreased probability of reporting being diagnosed with diabetes or high blood sugar, and respondents from Tierra del Fuego displaying a higher probability (OR = 1.54, 95% CI = 1.09 – 2.18).

**Figure 2:**
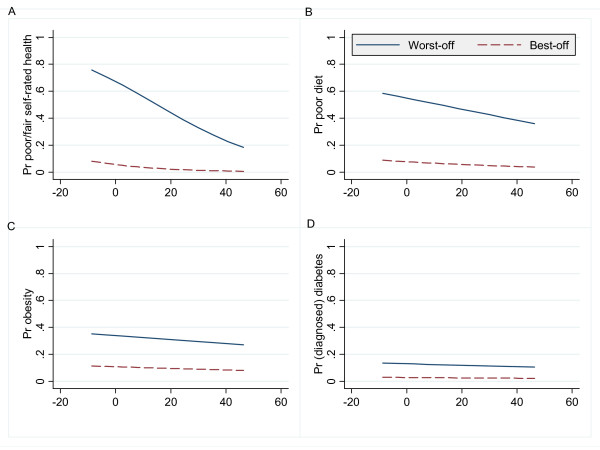
**ADI analysis of predicted probabilities by outcome measure**. *Notes*: Household monthly income (100s pesos, centred around the mean of 860 pesos) is shown on the x-axis of each graph. Pr refers to probability. Worst-off groups: Unemployed females in Jujuy, low education group and reliant on public sector healthcare (2A), Males in Santa Cruz, low education group and reliant on public sector healthcare (2B), Respondents from Tierra del Fuego, low education group (2C & 2D). Best-off groups: Employed males in La Pampa, high education group and with health insurance (2A), Females in Jujuy, high education group and with health insurance (2B), Respondents from Misiones, high education group (2C), Respondents from Jujuy, high education group (2D).

An ADI analysis of the logistic regression model is presented in figures 2A–D. Following the deprivation perspective, each figure plots the predicted probabilities (Pr) for the worst-case scenario (an ideal type based on the results of the logistic regression models). Following the inequality perspective, this is contrasted with the predicted probabilities for the best-case scenario (again, defined on the basis of the results from the logistic regressions).

Figure 2A shows the steepest pattern of the four graphs because the variable plotted on the x-axis (household income) was particularly influential in that specific regression model; the shallower lines depicted in figures 2B–D reflect the lesser significance of household income in those models. The difference between the lines in each graph is also important; these reflect the differential effects of the other independent variables in each model. Larger differences between the worst-off and best-off lines reflect larger inequalities.

## Discussion

Our analysis of data from Argentina's first nationally-representative survey of risk factors for chronic non-communicable diseases highlights the social patterning underlying self-reported health status, a common modifiable risk factor (unhealthy diet), an intermediate risk factor (obesity) and a chronic disease outcome (diabetes). Overall, 19.9% of the sample reported being in poor/fair health, 35.3% reported not eating any fruits or vegetables in five days of the week preceding the interview, 14.7% had a BMI of 30 or greater, and 8.5% indicated that a health professional had told them that they have diabetes or high blood pressure. As may be expected, these figures hide substantial variation. To explore that variation, we have utilized the ADI framework. A set of socioeconomic and demographic factors were considered as independent variables; educational attainment displayed the strongest explanatory power throughout the statistical models, followed by household income. More specifically, clear social gradients in self-reported health status, diet, obesity and diabetes were observed by educational attainment, even after controlling for other independent variables. Household income was strongly associated with self-reported health status and diet, but showed mixed effects with obesity and diabetes. Unmet basic needs displayed explanatory power in unadjusted models for self-rated health and diet but was not a significant predictor in models of obesity and diabetes. These findings suggest that measures of educational attainment may offer the most appropriate means to investigate social inequalities in health in countries like Argentina.

Our analyses also indicate the presence of significant provincial effects. This is displayed in the ORs associated with each province in the unadjusted models, and to a lesser extent, in the adjusted models. Congruent with a growing body of literature on the social determinants of health, these results suggest that *place *matters. Indeed, a key aspect of the ADI framework is that it works best with data than can be disaggregated not only by social categories, but by geography. The inclusion of dummy variables for provinces in our logistic regression models substantially influences the results. In models that account for socioeconomic and demographic factors, respondents from the northern provinces of Jujuy, Salta, and Tucumán display higher probabilities of reporting poor/fair health, but interestingly, they are *less likely *to have had an unhealthy diet in the week preceding the interview. Furthermore, residence in Jujuy is associated with a statistically significant decrease in likelihood of diabetes. This may reflect an underlying pattern wherein respondents from poorer provinces like Jujuy may be more likely to remain undiagnosed diabetics. Data to verify this interpretation are not available in the ENFR, but the existing literature on this topic supports this assertion. Chacra et al [20], in an influential overview of the burden of type 2 diabetes in Latin America, note that 30% of people with type 2 diabetes in the region are undiagnosed, and notably, that this figure rises to 90% in some rural areas (like Jujuy). A recent Pan American Health Organization report from Venezuela supports this assertion, noting that more than 40% of diabetics in that country may be undiagnosed and lack treatment [47]. Similar rates have been reported in Brazil [48]. In Canada, it is believed that undiagnosed cases of diabetes amount to one-third of all cases in the country [49]. Congruent findings from the United States suggests a notable social patterning to remaining undiagnosed [50], indicating that undiagnosed diabetes – which may manifest in skin infections, kidney problems, and vision problems and other preventable complications [51] – may be an important concerns from the perspective of equity in health. Future studies could build from the provincial data presented in the ENFR to examine the extent of undiagnosed, or hidden, diabetes in provinces like Jujuy. Combined with analysis of Jujuy's status in terms of the nutritional transition [52], such research could hold particularly useful policy implications.

These findings reflect myriad social, historical, and political factors that warrant further investigation. Future research could explore this patterning with the use of multilevel modelling [53-55], which would enable the inclusion of contextual factors (i.e., characteristics of the provinces) into the regression model. Such an analysis would contribute to a more nuanced epidemiological analysis, one informed by sociology and political economy. This would to a large measure overcome the limitations of a narrowly-focused individual-level risk factor epidemiology which often dominates discussions of chronic non-communicable diseases.

Application of the ADI framework enables a straightforward identification of the regions/groups worst-off for each outcome measure under study. We believe this has important policy implications, in that programs could be targeted to improve the standing of groups particularly burdened by risk factors or disease outcomes. Longitudinal analysis, based either on repeated cross-sectional surveys or panel surveys, could track changes over time to evaluate policies aiming to improve the situation of the worst-off. Such analyses would complement the growing literature on health and social justice [56]. The third step in the ADI framework, the inequality perspective, also holds key policy implications in the sense that ideal policies would not only improve the situation of the worst-off but would also narrow the gap between the worst-off and the best-off.

There are at least three limitations to the analyses presented in this paper. Firstly, the analysis is based entirely on self-reported measures. Granted, the ENFR instrument has been validated in Argentina [26], and is based on a robust and widely-accepted instrument originally developed by the World Health Organization [57]. However, despite validation of the survey instrument, the ENFR, like any other household survey, will suffer from social desirability [58] and under/over reporting of some data. In particular, we may expect an under-reporting of income among higher income groups. Indeed, previous research from Argentina suggests that this may be a problem [31,59], resulting in downwardly biased relationships between income and health outcomes. At the same time, error can also be expected in the income reporting of individuals involved in the informal economy. This may lead to an underestimate of the income of the poor and lower middle class. To address these issues, future research should utilize measures of wealth, rather than just income. The analyses are also limited in the sense that they do not explore interaction effects among the independent variables and do not incorporate contextual factors. Given the recognized importance of such factors as social determinants of health [15,60], this would be an appropriate next step in the analysis. Lastly, the analysis is limited by the fact that the ENFR is a cross-sectional survey. Longitudinal data would offer more insight into the dynamics underlying the social patterning of risk factors for chronic diseases. Alternatively, repeated cross-sectional waves of the ENFR, repeated every 3–5 years, would similarly yield information that could be analyzed using the ADI framework.

Overall, these analyses begin to highlight the social patterning of risk factors for chronic diseases in Argentina. Future analyses should delve deeper into this issue by examining other important risk factor measurements in the ENFR, including physical activity, cholesterol, tobacco, alcohol, and sexual health. Analyses could also attempt to link data from the ENFR with other datasets in the country, particularly surveys of living conditions, thus opening the way for more nuanced analytical approaches to investigating the social determinants of health in Argentina. Future work ought also to consider harmonizing selected variables from Argentina's ENFR with similar studies recently carried out in other countries from the Southern Cone. The results of that analysis could compare social gradients by income and educational attainment, or could utilize illness concentration curves [61,62] to map out inequities across regions and countries.

## Conclusion

Incorporating the ADI framework in population health research encourages a move towards a more comprehensive understanding of the underlying social patterning of chronic diseases. It can be used to highlight the variation embedded within national averages; as such, it encourages a social perspective on population health indicators that is particularly attuned to issues of inequity. It is promising tool for the evaluation of policies aiming to prevent or control chronic non-communicable diseases.

## Competing interests

The authors declare that they have no competing interests.

## Authors' contributions

FDM and BL conducted the data analysis. FDM wrote the first draft of the paper. All authors contributed to revising the manuscript and to the writing of the final draft. All authors have read and approved the final manuscript.
